# The influence of Epstein-Barr virus in the microenvironment of classic Hodgkin lymphoma: A focus on T-cells and macrophages

**DOI:** 10.1016/j.htct.2026.106535

**Published:** 2026-09-25

**Authors:** Ana Claudia Frota Melo, Valéria Buccheri, Jackson A. Barbosa, R.S. Azevedo, Mario L. Marques-Piubelli, MCN. Zerbini

**Affiliations:** a Hospital das Clínicas, Faculdade de Medicina, Universidade de São Paulo, São Paulo, SP, Brazil; b Department of Pathology and Genomic Medicine, Houston Methodist Hospital, Houston, TX, USA

**Keywords:** Hodgkin lymphoma, Tumor microenvironment, Epstein Barr virus, Follicular helper T-cells, Regulatory cells, Tumor macrophages

## Abstract

**Background:**

The prominent immune landscape in classic Hodgkin lymphoma is an essential tool to understand the pathogenesis of the disease.

**Objective:**

The aim of this study was to evaluate the influence of the Epstein-Barr virus on the frequency and spatial distribution of lymphoid T-cells and macrophages in the immune microenvironment.

**Methods:**

Pre-treatment lymph node biopsies of 54 Epstein-Barr virus-positive and 91 virus-negative cases of classic Hodgkin lymphoma were studied. Different immune subsets, such as macrophages, T-helper, T-follicular helper, T-regulatory, and T-cytotoxic cells, were evaluated by digital image analysis according to their position in areas close to or distant from neoplastic cells.

**Results:**

When compared to virus-negative cases, Epstein-Barr virus-positive classic Hodgkin lymphoma exhibited a higher prevalence of macrophages and CD8^+^ T-cells in both close and distant areas. Furthermore, CD4^+^ T-cells in distant areas and their subpopulations (T-regulatory and T-follicular helper) were diminished (p-values: CD68 close: p = 0.028, distant: p = 0.022; CD163 close p < 0.001, distant p = 0.003; CD8 close and distant p < 0.001; CD4 distant: p = 0.04). Clinically, Epstein-Barr virus-positive patients were older by a decade and had a higher stage of disease when compared to virus-negative cases. No associations of viral status with refractoriness to treatment, recurrence, or disease-related death were observed.

**Conclusions:**

Cases of Epstein-Barr virus-positive classic Hodgkin lymphoma are related to a cytotoxic predominance, a significant macrophage component, and a decrease in T-follicular helper and T-regulatory cell populations compared to virus-negative cases.

## Introduction

Classic Hodgkin lymphoma (cHL) is a neoplasm originating from germinal center B cells that exhibits distinct pathological features. Histologically, the tumor is characterized by scattered neoplastic Hodgkin and Reed-Sternberg (HRS) cells immersed within a prominent tumor microenvironment (TME) [1, 2, 3], which exhibits distinct clinicopathological features and a unique cytokine and chemokine profile [4, 5, 6, 7].

In cases of Epstein-Barr virus (EBV)-positive cHL, a characteristic antiviral response and T-cell genes, like *CXCL10* (*IP-10*), are enhanced [8], which suggests an important role of EBV in the modulation of the tumor microenvironment [9]. The EBV latent membrane protein 1 (LMP-1) promotes one of the EBV effects through its capacity to induce the secretion of immunosuppressive cytokines such as interleukin (IL)-6, IL-8, and IL-10 [10, 11, 12, 13]. T-cells and macrophage subsets have been explored in the TME of cHL but their roles in the outcomes of these patients is still controversial [14, 15, 16]. T-follicular helper (T_FH_) cells, implicated as one for the main drivers for germinal center development, are a distinct subtype within this group [17]. These cells participate in the generation of high-affinity B-cell receptors (BcRs) by promoting the somatic hypermutation, resulting a higher affinity for the antigen [17]. T-regulatory (Treg) cells are actively recruited by HRS cells, a process that is amplified in EBV-positive tumors. Tregs subsequently surround the tumor cells and are capable of suppressing effector T-cell responses [28,29]. Tumor-associated macrophages are present in high numbers in cHL and have been implicated in the pathogenesis of this disease; nevertheless, literature has shown controversial results on the role of these cells in the clinical behavior of cHL [15, 16, 17]. In this study, the interaction of neoplastic cells in cases of EBV-positive cHL and EBV-negative cHL with the TME was explored, focusing particularly on the influence of EBV on the T_FH_ cell subset and macrophages. An *In situ* approach, which preserves spatial relationships that are typically lost in traditional tissue analysis was utilized to capture these cellular changes within their native architecture.

### Objective

Considering the heterogeneity of cHL association with EBV, this study aims to evaluate the effects of the virus on the type, frequency, and topographic distribution of lymphoid cells and macrophages in the TME. The topographic distinction in areas close to (AC) and areas distant from (AD) the HRS cells aimed to evaluate the influence of the virus itself on cells in close contact with neoplastic cells, as opposed to more distant areas, under the effect of the interaction between the inflammatory cells themselves. This is the first *In situ* study of the T_FH_ cells in Hodgkin disease. Differences related to the impact of EBV on the cHL TME may justify a better understanding of heterogeneity in response to treatment and the investment in specific therapeutic strategies.

## Materials and methods

### *Patient selection*

This retrospective study included patients newly diagnosed with cHL between January 2004 and December 2014 at a single academic center. Excisional lymph node biopsies were evaluated, and the diagnosis and histological classification (nodular sclerosis, mixed cellularity, lymphocyte-rich, and lymphocyte-depleted) were confirmed by a hematopathologist. Clinicopathologic characteristics such as age, sex, presence of B symptoms, staging according to the Lugano 2014 classification [26], treatment response, relapse or refractoriness, overall survival, and death related to the disease were recorded by reviewing medical records. Patients with incomplete clinicopathologic data and any known immunosuppressive condition, including HIV-positive and non-available histological material, were excluded from the study. This study was approved by the institutional ethics review board of the Universidade de Sao Paulo (protocol number 1.629.293) and was conducted in accordance with the principles of the Declaration of Helsinki.

### *Selection of the areas of interest and tissue microarray preparation*

For each case, six regions of interest (ROIs) were randomly selected: three areas adjacent to HRS cells (AC) and three areas distant from them (AD). Following a full-slide review, AD regions were chosen from central areas completely devoid of HRS cells identified using a 10x objective. In contrast, AC regions were selected immediately adjacent to identifiable neoplastic (HRS) cells. From each donor paraffin block, six tissue cores, three from AC regions and three from AD regions, were extracted using a 1.0 mm needle. The cores were then arrayed into a recipient paraffin block at predefined coordinates using a tissue microarrayer (Beecher Instruments, Inc., Sun Prairie, WI, USA). This describes the *in-situ* methodology, which was selected to enable the targeted analysis of specific tumor regions. By preserving the tissue's spatial architecture, this approach allowed the characterization of local cellular composition and assess its direct impact on tumor biological behavior.

### *Immunohistochemistry and In situ hybridization*

Primary antibodies were used to evaluate the cell subsets, as follows: CD3, CD4, CD8, CXCR-5, PD-1, Foxp3, CD68, CD163 and LMP1 (Table 1). All immunohistochemical reactions were performed manually on paraffin-embedded sections from the tissue microarray (TMA) blocks. Signal amplification was carried out using the Novolink Polymer system, followed by visualization with a DAB solution (3,3′-diaminobenzidine tetrahydrochloride and dimethyl sulfoxide – Sigma, USA). Slides were counter-stained with hematoxylin, dehydrated, and cover slipped. Adequate positive and negative controls were carried out. *In situ* hybridization for Epstein-Barr virus-encoded RNA (EBER) was performed using the INFORM EBER Probe on the BenchMark ULTRA automated stainer with the ISH Detection Kit (Ventana Medical Systems), according to the manufacturer’s protocol.

**Table 1:** Antibodies used and their specifications.

**Antigen**	**Manufacturer**	**Code**	**Clone**	**Titer**	**Retrieval method**	**Target Cell**
**CXCR5**	ABCAM	Ab46218	Polyclonal	1:1500	EDTA, pH 8	T_FH_ Cell
**PD1**	ABCAM	Ab52587	NAT105	1:50	TRIS-EDTA, PH9	T_FH_ Cell
**CD68**	DAKO	M0876	PG-M1	1:2500	Citrate, pH 6	Macrophage
**CD163**	LEICA NOVOCASTRA	NCL-l-CD163	10D6	1:300	TRIS- EDTA, pH 9	Macrophage
**CD3**	LEICA NOVOCASTRA	NCL-l-CD3–565	LN10	1:1500	Citrate, pH 6	T Cell
**CD4**	LEICA NOVOCASTRA	NCL-LCD4–368	4B12	1:50	TRIS- EDTA, H 9	T-Helper lymphocyte
**CD8**	DAKO	M7103	C8/144B	1:400	EDTA pH 8	T-cytotoxic lymphocyte
**FOXP3**	Epitomics	EO01–2305	EP340	1:100	TRIS- EDTA, pH 9	T Regulatory lymphocyte
**EBV**	ABCAM	Ab78113	CS 1–4	1:800	TRIS- EDTA, pH 9	LMP1/LMP2

EBV: Epstein Baar virus; LMP1/LMP2: EBV Latent Membrane Protein 1 and 2; EDTA: ethylenediaminetetraacetic acid.

### Immunoexpression analysis and quantification

TMA spots were considered valid for immunohistochemical analysis if at least 50% of the spot area contained tumor tissue. All slides were digitized using a Zeiss Laser Scanning microscope (Germany) and Pannoramic Viewer (version 1.15; 3DHISTECH, Hungary) with a 40x objective. High-resolution images were captured at 400x magnification for morphometric analysis.

Quantitative analysis of CD3, CD4, CD8 Figure 1, CD68 and CD163 (Figure 2) was performed using Image-Pro Plus 4.5 software (MediaCybernetics, USA). These markers were evaluated based on the proportion of immunoreactive area relative to the total tissue area.

**Fig. 1:**
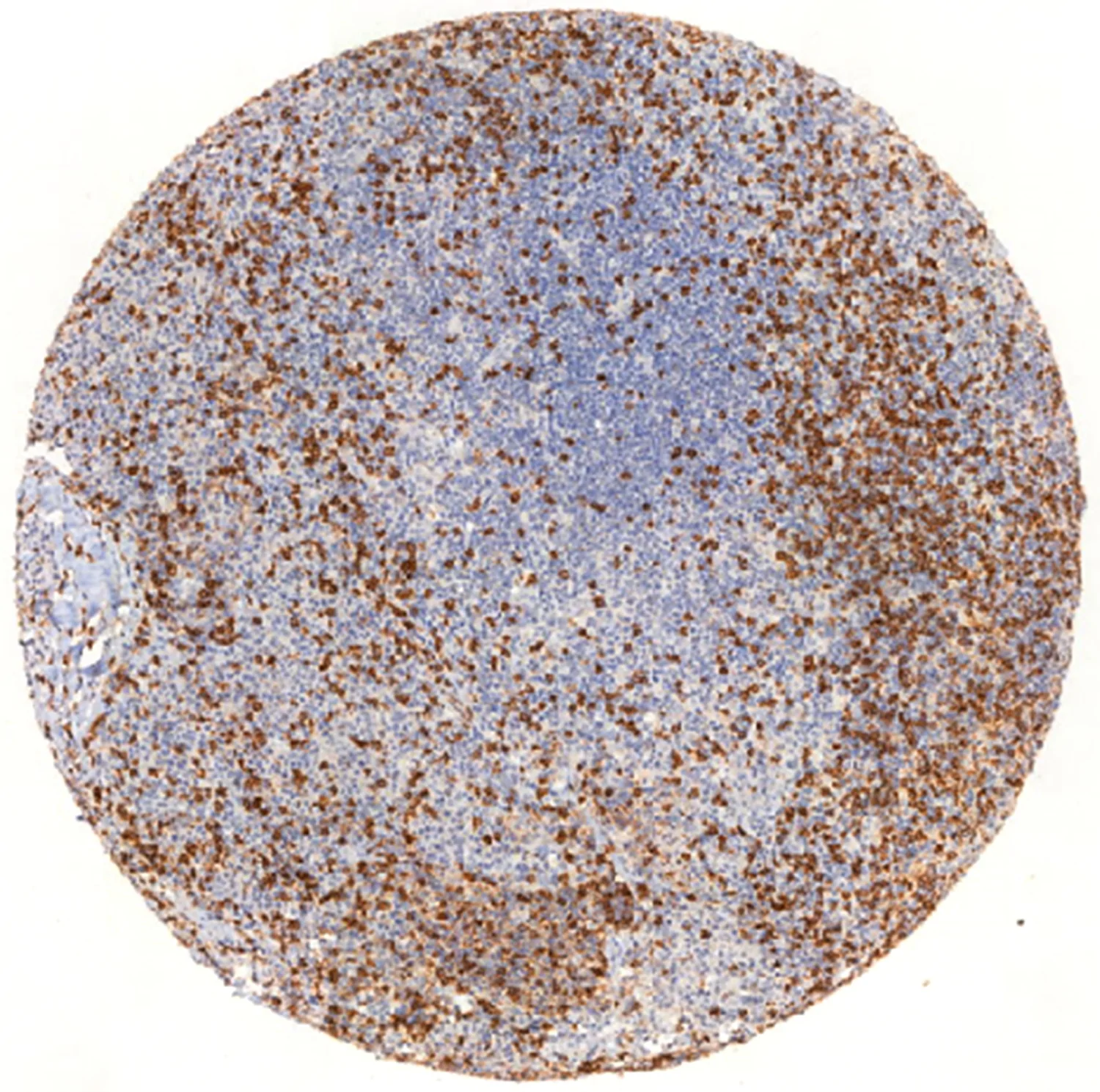
Tissue microarray CD8 immunomarker spot (380x).

**Fig. 2:**
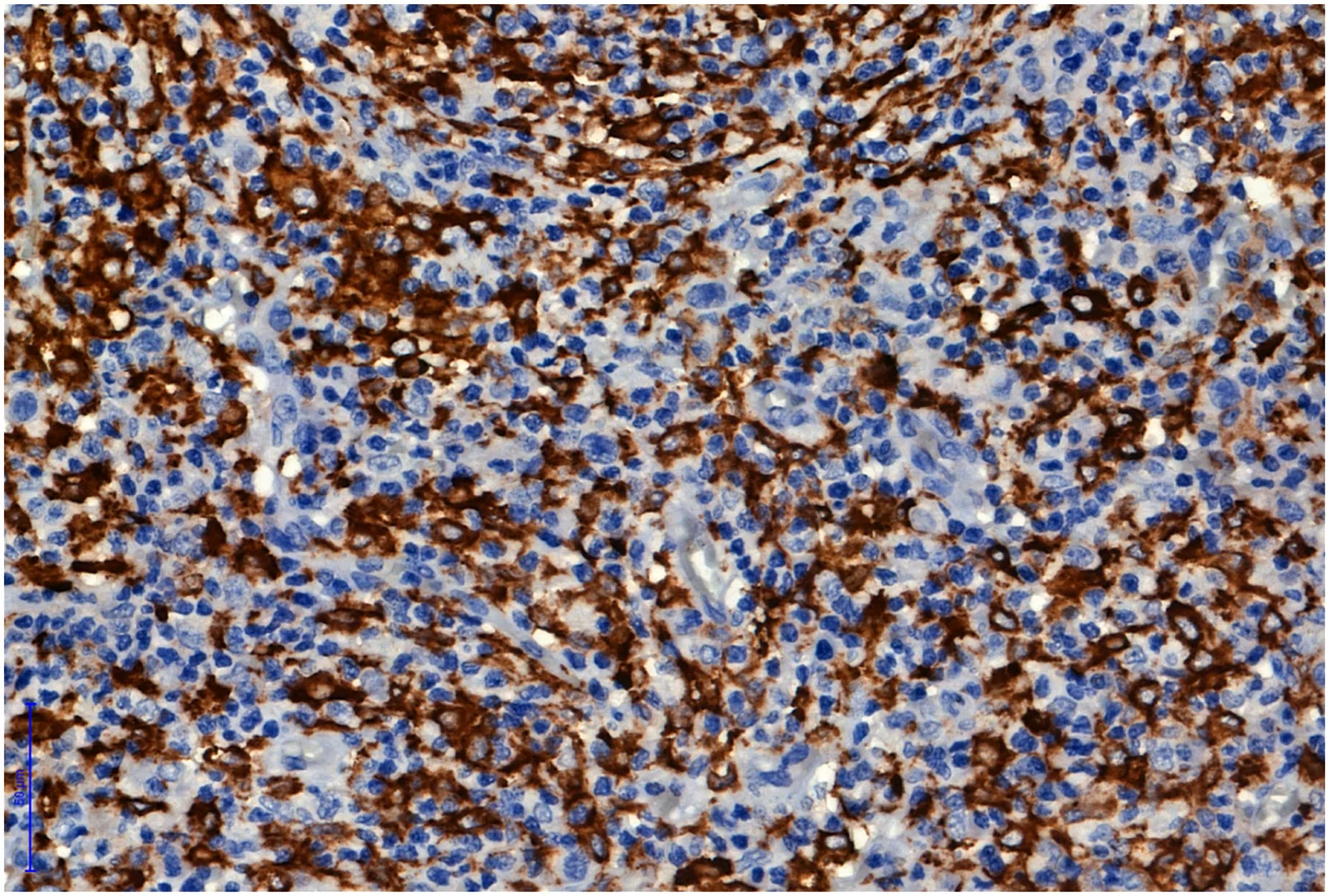
Tissue microarray spot - immunoexpression of CD163 (2000x).

For CXCR-5 (Figure 3), PD-1 (Figure 4), and Foxp3, analysis was done by counting the number of positive lymphocytes per high-power field (400x) across the entire spot. The means of the three AC spots and the three AD spots were calculated for each case.

**Fig. 3:**
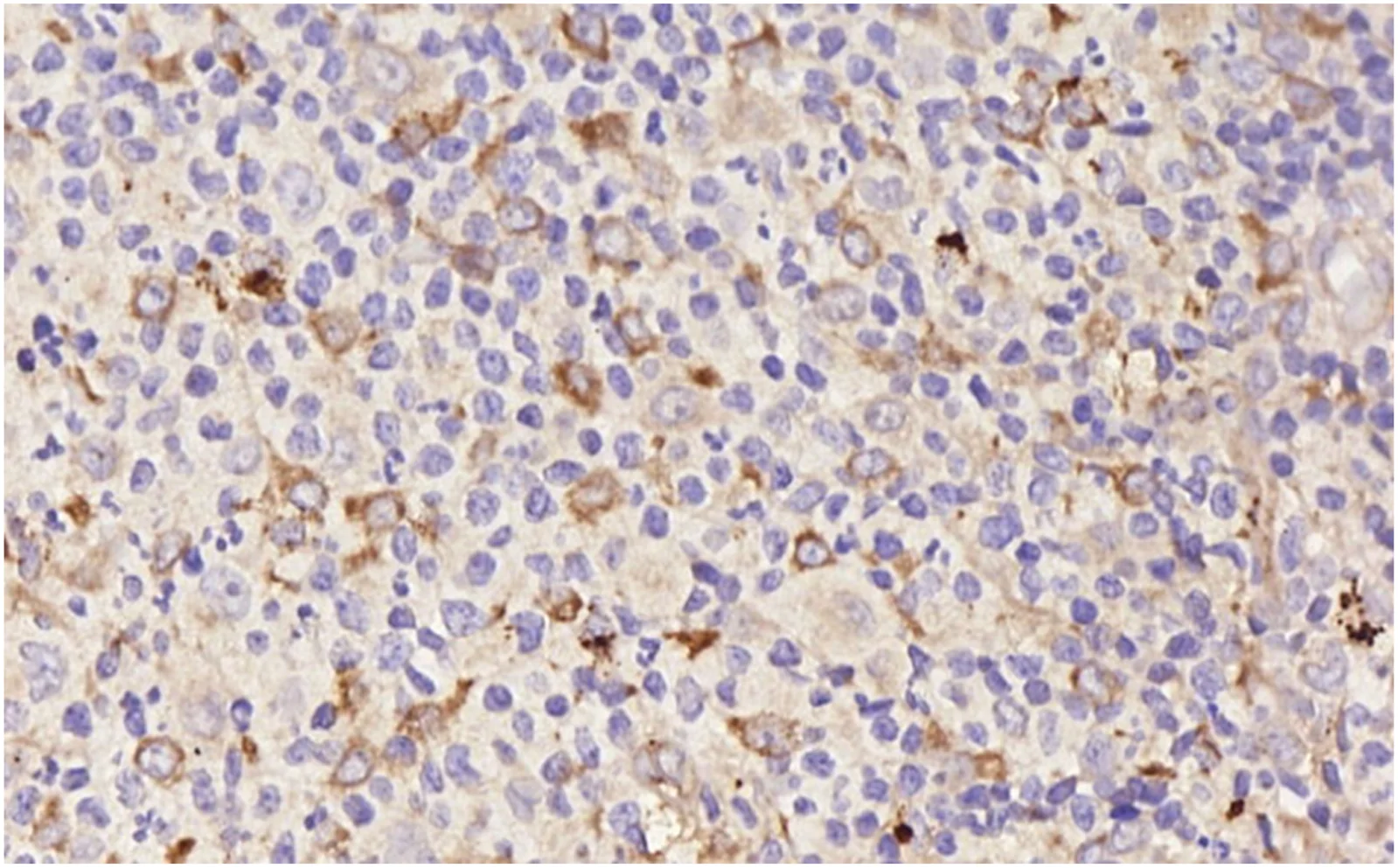
Tissue microarray spot - immunoexpression of CXCR-5 (2000x).

**Fig. 4:**
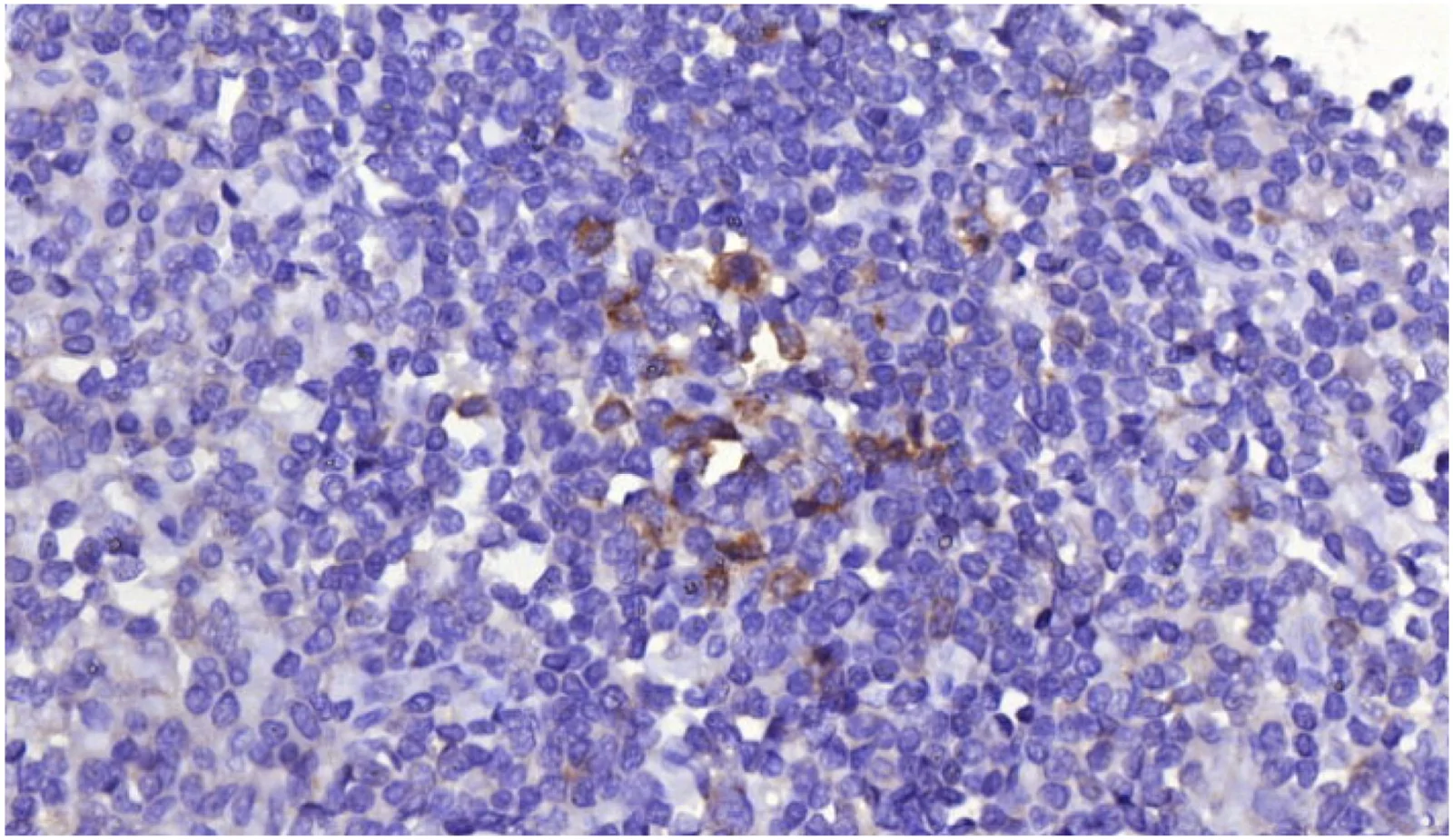
Tissue microarray PD1 spot (2000x).

A case was classified as EBV-positive if HRS cells demonstrated positivity for at least one of the two markers (EBER or LMP1). This dual-marker approach was adopted to maximize detection sensitivity, as each technique captures different aspects of EBV biology. EBER-*In situ* hybridization (ISH) detects viral RNA transcripts and is considered the gold standard for EBV detection. Cases with inconclusive LMP1 results and negative EBER-ISH were classified as EBV-negative, as EBER-ISH is the established reference standard. Cases with inconclusive LMP1 results and positive EBER-ISH were classified as EBV-positive based on the EBER result. The concordance between EBER-ISH and LMP1 across all 145 cases is detailed in Supplementary Table 1.

### Statistical analysis

The association between categorical variables was evaluated using chi-square, while the *t*-test and Kruskal-Wallis test were used for continuous variables as appropriate. A p-value ≤0.05 (two-tailed) was considered statistically significant. The statistical analyses were performed using Minitab Statistical Software (Minitab® Statistical Software – 18th Version, State College, Pennsylvania).

## Results

### *Baseline characteristics*

A total of 145 lymph node biopsies were retrieved. Fifty-four were EBV-positive (female: n = 28; male: n = 26) and 91 EBV-negative (female: n = 52; male: n = 39) (p = 0.536). Initially, the patient profile was analyzed according to the different EBV status (Table 2). The mean age of patients with EBV-positive cHL was a decade higher than those with EBV-negative tumors (40.8 years *vs.* 31.1 years respectively; p = 0.001). Among histologic subtypes, nodular sclerosis was the most common and was diagnosed in 114 patients (79%); mixed cellularity subtype was the second most prevalent subtype, present in 17 cases, 13 of which were EBV-positive cHL cases (p = 0.03); the remaining cases were described as lymphocyte rich in eight patients and as inconclusive in six cases. Regarding tumor staging, the group of EBV-negative cHL presented a similar distribution between low and high stages. In contrast, the EBV-positive cHL group showed more patients with higher stages (p = 0.005). The presence of B symptoms, and the occurrence of refractoriness, relapse and death were also evaluated, but these associations did not show any statistical significance. All these results are summarized in Table 2.

**Table 2:** Clinical characteristics and histological subtype in different EBV status.

Ebv Status	**Total (%)**	**EBV-positive cHL**	**EBV-negative cHL**	**p-value**
Clinical Parameter				
**n (%)**	145 (100)	54 (37)	91 (63)	
**Age (mean ± SD)**	34.7 ± 15.6	40.8 ± 17.9	31.1 ± 12.9	0.001
**Sex (F/M)**	80/65 (55/45)	28/26 (19/18)	52/39 (36/27)	0.536
**Histological Type (%) (N=145)**				
**MC**	17 (12)	13 (76)	4 (24)	0.003
**LR**	8 (5)	4 (50)	4 (50)	
**NE**	114 (79)	35 (31)	79 (69)	
**LD**	0	0	0	
**Inconclusive**	6 (4)	2 (4)	4 (4)	
**B Symptoms (%) (N=145)**				
**Present**	110 (76)	42 (29)	67 (46)	0.448
**Absent**	35 (24)	11 (8)	24 (17)	
**Stage (%) (N=144)**				
**I/II**	62 (43)	15 (10)	47 (33)	0.005
**III/IV**	82 (57)	38 (27)	43 (30)	
**Refractoriness (%) (N=132)**				
**Present**	21 (16)	5 (4)	16 (12)	p = 0.118
**Absent**	111 (84)	46 (35)	65 (49)	
**Relapse (%) (N=145)**				
**Present**	34 (23)	8 (5)	26 (18)	0.053
**Absent**	111 (77)	46 (32)	65 (45)	
**Death (%) (N=145)**				
**Absent**	128 (88)	46 (32)	82 (57)	0.379
**Present**	17 (12)	8 (5)	9 (6)	

cHL: Classic Hodgkin lymphoma; SD: Standard deviation; F: Female; M: Male; MC: Mixed cellularity; LR: Lymphocyte rich; NE: Nodular sclerosis; LD: Lymphocyte depletion.

### *T-cell markers*

Of the T-cell markers (Table 3, Table 4), Foxp3 and CD4 presented a lower immunostaining score in EBV-positive tumors: Foxp3 in AC (p = 0.033) and CD4 in AD (p = 0.04). Meanwhile, CD8 revealed a higher proportion of cells in EBV-positive tumors, in both AC and AD (p < 0.001) (Table 3). Regarding T_FH_ cells, CXCR-5 showed a lower number of positive cells in EBV-positive tumors in both AC and AD (p = 0.001 and p = 0.002, respectively). PD-1 expression did not differ significantly between the EBV-positive and EBV-negative groups, nor were significant differences observed for FOXP3 expression in AD, CD4 expression in AC, or CD3 expression in either AC or AD regions (Table 4).

**Table 3:** T Cells and EBV status in different tumor areas (% positive areas).

		**CD3 (Median)**			**CD4 (Median)**			**CD8 (Median)**		
**Areas according to the relationship with the HRS cells**		n	AC	AD	n	AC	AD	n	AC	AD
**EBV Status**	**Positive**	54	20.71%	22.01%	51	7.36%	5.86%	54	11.57%	9.42%
	**Negative**	91	22.98%	22.45%	91	12.66%	12.11%	91	4.49%	5.76%
**p-value**			0.537	0.702		0.054	0.04		<0.001	<0.001

HRS: Hodgkin and Reed-Sternberg cells; AC: areas close to HRS cells; AD: areas distant from HRS cells.

**Table 4:** T_REG_ cells_,_ T_FH_ Cells and EBV status in different tumor areas (immunopositive cells/high power field* - 0.332 mm^2^).

		**CXCR-5 (Median)**			**FoxP3 (Median)**			**PD-1 (Median)**		
**Areas according to the relationship with the HRS cells**		Nn	AC	AD	n	AC	AD	n	AC	AD
**EBV Status**	**Positive**	54	14	14	31	23	16	24	13.5	61
	**Negative**	91	21	22	55	38	23	35	13	23
**p-value**			0.001	0.002		0.033	0.082		0.66	0.44

T_REG_: T-regulatory cells_,_ T_FH_: T-follicular helper cells; HRS: Hodgkin and Reed-Sternberg cells; AC: areas close to HRS cells; AD: areas distant from HRS cells.

### *Macrophages*

Macrophage analysis of both markers, CD163 and CD68 (Table 5), showed higher scores in EBV-positive patients in the two areas (CD163: AC, p < 0.001, AD, p = 0.03; CD68: AC, p = 0.028, AD, p = 0.022). These results are summarized in Table 5.

**Table 5:** Macrophages and Epstein-Barr virus (EBV) status in different tumor areas (% positive area).

		**CD163 (Median)**			**CD68 (Median)**		
**Areas according to the relationship with the HRS cells**		n	AC	AD	n	AC	AD
**EBV Status**	**Positive**	52	21.5%	22.40%	54	3.02%	2.68%
	**Negative**	91	12.8%	13.6%	91	2.04%	2.02%
**p-value**			<0.001	0.003		0.028	0.022

HRS: Hodgkin and Reed-Sternberg cells; AC: areas close to HRS cells; AD: areas distant from HRS cells.

## Discussion

Despite the strong immunomodulatory effects of EBV, most studies analyzing the TME in cHL did not consider the influence of the virus on the immune landscape of these neoplasms. In the present study, although the microenvironment composition of areas immediately adjacent to neoplastic cells was similar to that of areas distant from HRS cells within cases sharing the same viral status, EBV contributes to modulating the microenvironment in Hodgkin lymphoma, with EBV-positive cases exhibiting distinct characteristics compared to EBV-negative cases. The results of this study showed that EBV status had a greater influence on microenvironment composition than proximity to the neoplastic cells.

In this study, the mean age of patients with EBV-positive cHL was 40.8 years, almost a decade older than the EBV-negative patients (31.1 years), which agrees with the findings by Campos et al. [2] and is similar to what is seen in developed countries. A possible explanation for this observation is immunosenescence, the age-related decline in T-cell-mediated immunity, which permits latent EBV-infected B cells to evade cytotoxic immune surveillance and undergo malignant transformation [3]. In this study, EBV-positive tumors were significantly associated with higher clinical stages compared to EBV-negative tumors, although the prognostic role of EBV in cHL is controversial in the literature. Consistent with the study by Lee et al. [4], no association was observed in the present study between viral status and conventional treatment refractoriness, relapse, or mortality. On comparing EBV-positive and EBV-negative cHL, the immunophenotype of HRS cells is identical. Similar to previous studies, a higher prevalence of the mixed cellularity subtype in EBV-positive cHL was demonstrated in the present cohort (Table 2) [5]. This finding reflects the differences in the microenvironment of these two groups of cHL, which is probably related to viral effects on the immune system.

While it has been shown that most cases of cHL are characterized by the unbalanced production of T_H2_ cytokines and chemokines [18], the presence of EBV favors a T_H1_ reaction [8]. Similarly, previous molecular data demonstrated that EBV-positive cHL tumors are characterized by a prominent gene signature involving innate immunity and antiviral responses [9]; these findings are corroborated in the present study by the *In situ* demonstration of a dominant population of CD8^+^ cytotoxic lymphocytes and CD68^+^/CD163^+^ macrophages in the TME of EBV-positive tumors when compared to EBV-negative cases. Elevated protein expression of CD68 and CD163 in EBV-positive cases has also been demonstrated in previous studies [10, 11, 12]. Furthermore, Chetaille et al. [9] reported the upregulation of macrophage-associated genes, including *CXCL9, CXCL10, STAT1*, and *cathepsins*, in EBV-positive cHL tumors [9].

Different approaches have been used to evaluate macrophages in cHL. Barros et al. [10] quantified cells per mm², whereas Kamper et al. [11] assessed immunoexpression by the percentage of positive cells, similar to the present methodology. The predominance of CD8^+^ T cells in EBV-positive patients may correlate with M1 macrophages, which adopt a pro-inflammatory phenotype, promote TH1 responses, and enhance tumor cell killing. Together, these findings support an antiviral immune response directed against EBV proteins expressed in HRS cells, as was proposed by Barros et al. [13]. Recent studies have also highlighted the potential prognostic impact of CD8^+^ T-cell density within the TME, with higher CD8^+^ T-cell infiltration correlating with enhanced responsiveness to chemotherapy [27].

T_FH_ cells, which express markers such as CXCR5 and PD-1, were also examined in this study. CXCR5 is the receptor for chemokine ligand 13 (CXCL13) produced by follicular dendritic cells; it guides B cells into germinal centers to promote T–B cell interactions. In humans, CXCR5, encoded by the *CXCR5* gene, is considered a defining T_FH_ marker [19]. Interest in CXCR5 has expanded beyond immunobiology: CAR-T cells engineered to target CXCR5 have shown high avidity *in vitro*, suggesting potential therapeutic applications [20].

In the present study, EBV-positive tumors showed fewer CXCR5^+^ T_FH_ cells in the TME compared with EBV-negative tumors. Given the role of T_FH_ in germinal center formation [17], reduced numbers could impair surveillance of aberrant B-cell clones undergoing somatic hypermutation and class switching, thereby favoring the persistence of EBV-altered cells. EBV is known to support lymphomagenesis by allowing B-cell survival even without a functional BcR, with LMP2a substituting for BcR signaling and enabling immortalization [17]. The biological importance of T_FH_ cells is further underscored by T_FH_-derived neoplasms such as angioimmunoblastic T-cell lymphoma, in which reactive EBV-positive HRS-like B cells often appear in the TME alongside neoplastic T_FH_ cells [21]. Inspired by this parallel, we explored the reverse situation: the interaction between neoplastic B cells in cHL and reactive T_FH_ cells in their microenvironment.

This study also evaluated Treg cells in the TME but found no significant differences between EBV-positive and EBV-negative tumors. This contrasts with findings from Pavlovic et al. [22], Morales et al. [23], and Assis et al. [24], likely reflecting methodological variability. For example, Pavlovic used the Santa Cruz FoxP3 clone with CD4 double-staining, Morales assessed gene expression by reverse transcription quantitative polymerase chain reaction (RT-qPCR), and Assis quantified FoxP3 using Aperio ImageScope with BD Biosciences antibodies. These differences highlight the need for a more standardized approach to FoxP3 evaluation in cHL.

We acknowledge several limitations in this study: first, the artificial distinction between adjacent and distant regions, which was mitigated by spatial *In situ* analysis, a methodology uniquely suited to provide insight into these microenvironmental spatial relationships. While the strong predominance of the nodular sclerosing subtype in the present cohort could confound microenvironment analyses, it accurately reflects the real-world epidemiology of the disease. Whilst most of the recent studies on cHL microenvironment focus on its relationship with PD-L1, this study focused on inflammatory cells and their relationship with EBV. Although PD-1 and CXCR5 were both evaluated in the current panel, single immunohistochemical staining for each marker may also label other lymphoid subpopulations. Therefore, results concerning T_FH_ cells should be interpreted with caution, and confirmation with multiplex or dual-staining techniques would further strengthen these findings. We also know that classical immunohistochemistry is not as good as multiplex technologies for this kind of analysis, which is an exciting perspective for future studies.

## Conclusion

In summary, EBV-positive HRS cells trigger strong immunogenicity, resulting in a tumor microenvironment distinct from EBV-negative cHL. This distinction may have therapeutic relevance, particularly when considering combined treatment regimens. The findings of this study also raise the hypothesis that T_FH_ cells may contribute to EBV-positive cHL lymphomagenesis, a possibility that warrants further validation. Exploring this association could deepen our understanding of cHL pathogenesis and uncover new therapeutic targets. Finally, the complex interplay of TME components, together with the limited specificity of inflammatory markers, underscores the need for advanced *In situ* studies using multiplex immunohistochemistry or immunofluorescence [25].

## Funding

This research did not receive any specific grant from funding agencies in the public, commercial, or not-for-profit sectors.

## Credit authorship contribution statement

Ana Claudia Melo: Conceptualization, Methodology, Formal Analysis, Investigation, Data Curation, Writing- Original Draft, Review and Editing, Project Administration; Valeria Buccheri: Resources; Jackson Barbosa: Data curation; Raymundo Soares Azevedo: Formal Analysis; Mario L Marques-Piubelli: Data curation, Writing - Review and Editing; Maria Claudia Nogueira Zerbini: Conceptualization, Methodology, Writing - Review and Editing, Supervision, Project Administration. All authors commented on previous versions of the manuscript. All authors also read and approved the final manuscript.

## Ethics approval and consent to participate

The need for informed consent was waived by the ethics committee of the Universidade de Sao Paulo, because of the retrospective nature of the study, guarantee of confidentiality and no additional harm to the patient. All protocols were approved by the Comite de ética e Pesquisa da Universidade de Sao Paulo and were conducted in accordance with their regulations and guidelines. Protocol numer 1.629.293

## Data availability

The data that support the findings of this study are available from the corresponding author upon reasonable request

## Conflicts of interest

The authors declare that they have no known competing financial interests or personal relationships that could have appeared to influence the work reported in this paper.

## Appendix Supplementary materials

Image, application 1
