# Supplementary material for: The influence of Epstein-Barr virus in the microenvironment of classic Hodgkin lymphoma: A focus on T-cells and macrophages
Source: Hematol Transfus Cell Ther. 2026 Sep 25;48(4):106535. doi: 10.1016/j.htct.2026.106535 (PMC13634430; doi:10.1016/j.htct.2026.106535)
Supplement: Supplementary file 1 [file mmc1.docx]

**Supplementary Table 1:** concordance between Epstein-Barr virus-encoded RNA- In Situ Hybridization (EBER-ISH) and EBV Latent Membrane Protein 1 (LMP1) across all 145 cases

| **EBV Classification** | **EBER-ISH** | **LMP1 IHC** | **n (%)** | **Interpretation** |
| --- | --- | --- | --- | --- |
| EBV-Positive | Positive | Positive | 40 (27.6%) | Concordant positive; EBV latency type II/III |
| EBV-Positive | Positive | Negative/ Inconclusive | 11 (7.6%) | EBV latency type I (EBER expressed, LMP1 absent) |
| EBV-Positive | Negative | Positive | 3 (2.1%) | Discordant; possible EBER RNA degradation in archival tissue |
| EBV-Negative | Negative | Negative | 26 (17.9%) | Concordant negative |
| EBV-Negative | Negative | Inconclusive | 65 (44.8%) | EBER gold standard negative; LMP1 technically inconclusive |
| **Total** |  |  | **145 (100%)** |  |

EBV: Epstein-Barr virus
